# Supplementary material for: Tapasin assembly surveillance by the RNF185/Membralin ubiquitin ligase complex regulates MHC-I surface expression
Source: Nat Commun. 2024 Oct 1;15:8508. doi: 10.1038/s41467-024-52772-x (PMC11445256; doi:10.1038/s41467-024-52772-x)
Supplement: Supplementary file 1 — Supplementary Information [file 41467_2024_52772_MOESM1_ESM.pdf]

Supplementary Information

Tapasin assembly surveillance by the RNF185/Membralin ubiquitin ligase complex regulates MHC-I surface expression

Michael L. van de Weijer[1], Krishna Samanta[1,8], Nikita Sergejevs[1,8], LuLin Jiang[2,3], Maria Emilia Dueñas[4,5], Tiaan Heunis[1,6], Timothy Y. Huang[2], Randal J. Kaufman[2], Matthias Trost[4], Sumana Sanyal[1], Sally A. Cowley[1,7], Pedro Carvalho[1]

[1] Sir William Dunn School of Pathology, University of Oxford, South Parks Road, Oxford, OX1 3RE, UK
[2] Degenerative Diseases Program, Genetics and Aging Research Center, Sanford Burnham Prebys Medical Discovery Institute, La Jolla, CA 92037, USA
[3] Current address: Altos Labs-Bay Institute of Science, Redwood City, CA, USA
[4] Biosciences Institute, Newcastle University, Framlington Place, Newcastle upon Tyne, NE2 4HH, UK
[5] Current address: Telethon Kids Institute, Perth, Nedlands WA 6009, Australia
[6] Current address: Immunocore Ltd, 92 Park Drive, Abingdon, OX14 4RY, UK
[7] James and Lillian Martin Centre for Stem Cell Research, Sir William Dunn School of Pathology, University of Oxford, Oxford, United Kingdom
[8] These authors contributed equally to this study

Correspondence should be sent to:
Pedro Carvalho (pedro.carvalho@path.ox.ac.uk; phone: +44 1865 618 654)

This PDF file includes:
Supplementary Fig. 1-4
Supplementary Table 1
Supplementary References

Supplementary Figure 1. RNF185/MBRL-deficient cells display elevated levels of the MHC-I chaperone TPSN.

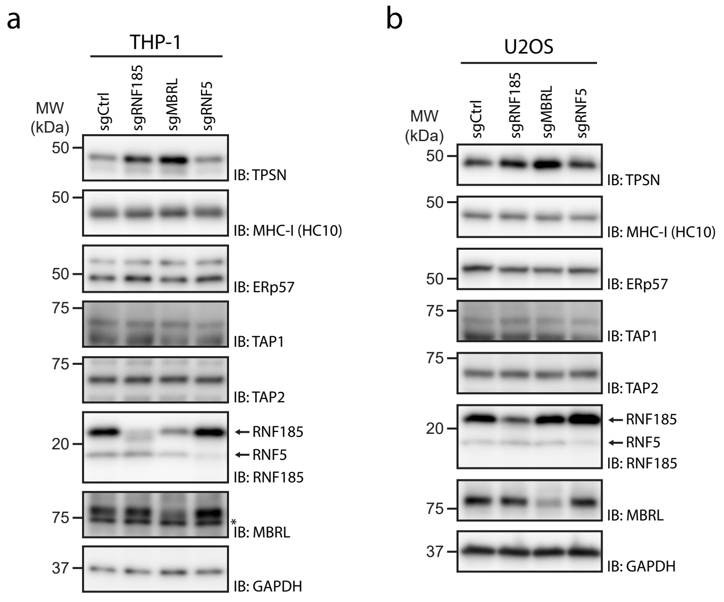

Supplementary Figure 1. RNF185/MBRL-deficient cells display elevated levels of the MHC-I chaperone TPSN.

(a, b) TPSN protein levels are increased in RNF185 and MBRL depleted (a) THP-1 and (b) U2OS cells. Extracts from parental, RNF185, MBRL, and RNF5 depleted cells were analysed by SDS-PAGE and immunoblotting with the indicated antibodies. The asterisk (*) indicates a non-specific background band.

Supplementary Figure 2. TPSN is a substrate of the RNF185/MBRL complex.

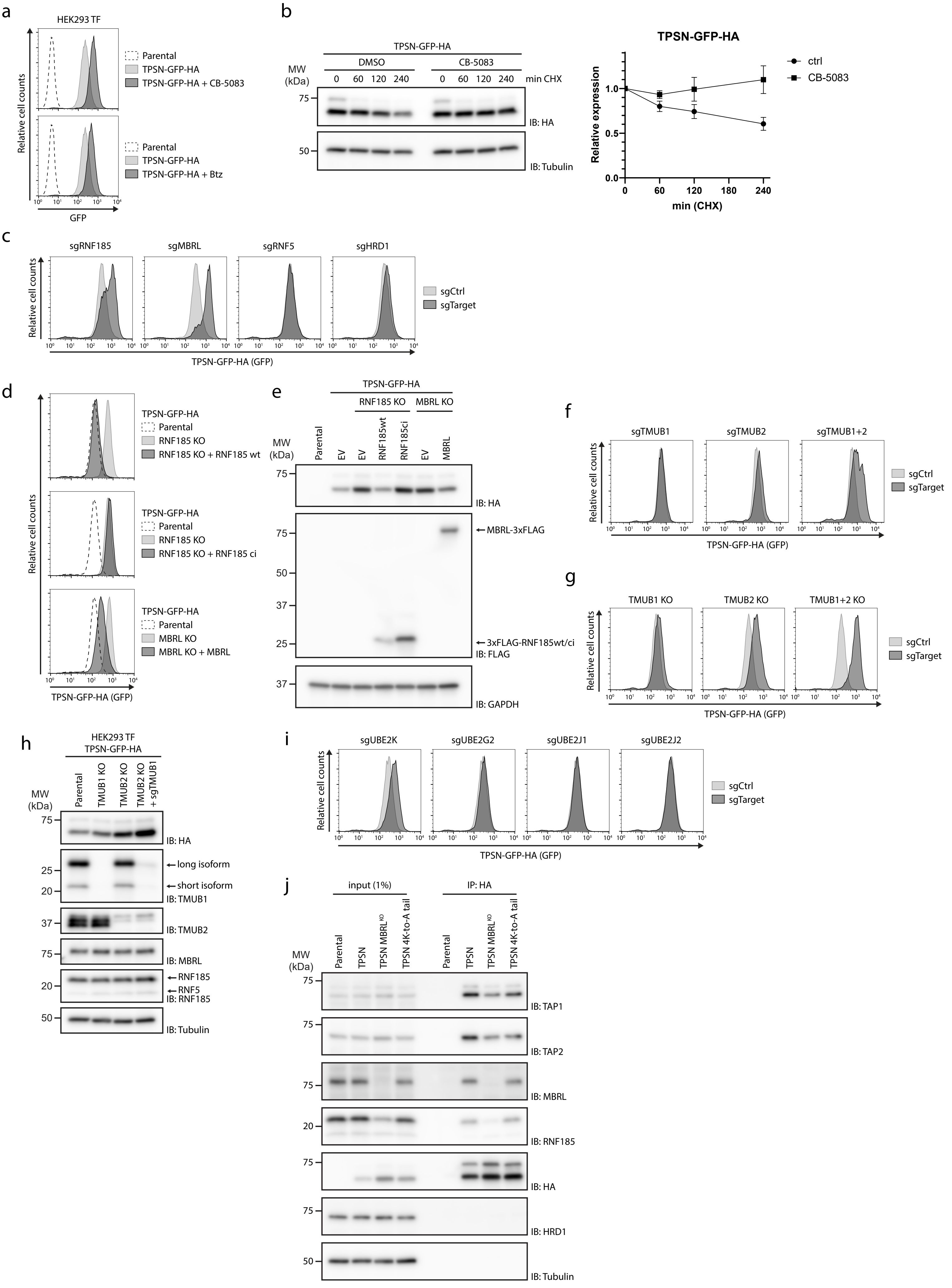

Supplementary Figure 2. TPSN is a substrate of the RNF185/MBRL complex.

(a) TPSN is stabilized upon p97 and proteasome inhibition. Flp-In T-Rex HEK293 cells expressing TPSN-sfGFP-3xHA were treated with the p97 inhibitor CB-5083 (2.5 μM) or the proteasome inhibitor Bortezomib (Btz; 500 nM) for 4 hours. TPSN-sfGFP-3xHA levels were assessed by flow cytometry (based on GFP fluorescence). Parental cells were included as control.

(b) Turnover of TPSN was analysed in Flp-In T-Rex HEK293 cells expressing TPSN-sfGFP-3xHA in the absence or presence of the p97 inhibitor CB-5083 (2.5 μM) after inhibition of protein synthesis by cycloheximide (CHX). Cell extracts were analysed by SDS-PAGE and immunoblotting. Quantifications of 3 independent experiments are shown in the graph below as mean; error bars represent the standard deviation.

(c) TPSN is stabilized in RNF185 and MBRL depleted cells. Flp-In T-Rex HEK293 cells expressing TPSN-sfGFP-3xHA were transfected with plasmids containing a control sgRNA or sgRNAs targeting RNF185, MBRL, RNF5 or HRD1. TPSN-sfGFP-3xHA levels were assessed by flow cytometry (based on GFP fluorescence).

(d, e) Clonal RNF185 KO Flp-In T-Rex HEK293 cells expressing TPSN-sfGFP-3xHA were transduced to stably express wildtype RNF185 or catalytic inactive RNF185. MBRL KO cells were transduced to stably express MBRL. TPSN-sfGFP-3xHA levels were assessed by (d) flow cytometry (based on GFP fluorescence) and (e) immunoblotting.

(f, g, h) TMUB1 and TMUB2 are essential for TPSN degradation. (f) Flp-In T-Rex HEK293 cells expressing TPSN-sfGFP-3xHA were transfected with plasmids expressing a control sgRNA or sgRNAs targeting TMUB1, TMUB2, or both. (g) From these, clonal TMUB1, TMUB2, and TMUB1+2 KO lines were generated. TPSN-sfGFP-3xHA levels were assessed by flow cytometry (based on GFP fluorescence) or (h) immunoblotting.

(i) The E2 UBE2K is involved in the degradation of TPSN. Flp-In T-Rex HEK293 cells expressing TPSN-sfGFP-3xHA were transfected with plasmids containing a control sgRNA or sgRNAs targeting UBE2K, UBE2G2, UBE2J1, or UBE2J2. TPSN-sfGFP-3xHA levels were assessed by flow cytometry (based on GFP fluorescence).

(j) Parental, TPSN-sfGFP-3xHA, MBRL KO TPSN-sfGFP-3xHA, and 4K-to-A tail TPSN mutant expressing cells were lysed in buffer containing 1% DMNG, after which TPSN was immunoprecipitated using anti-HA beads. Proteins were eluted and subjected to SDS-PAGE, followed by immunoblotting with the indicated antibodies.

Supplementary Figure 3. The RNF185/MBRL ERAD complex is not essential for MC80-induced ERAD of TPSN.

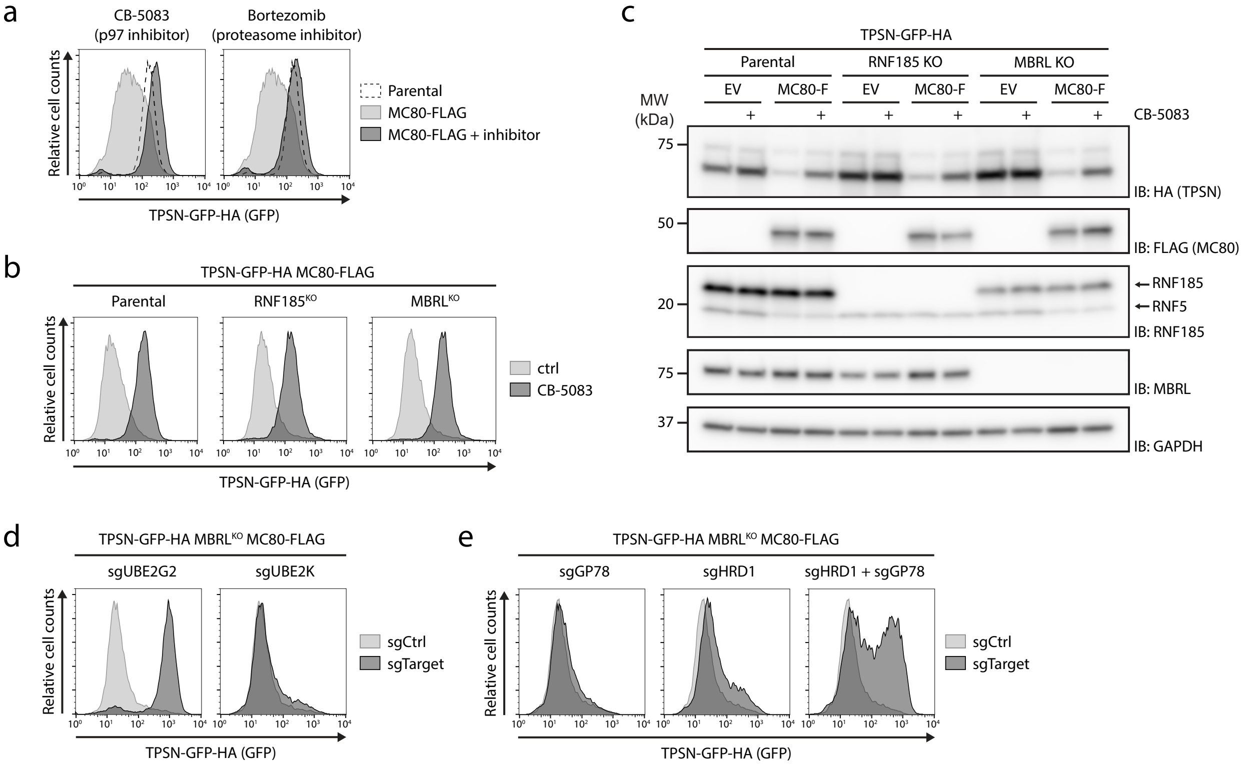

Supplementary Figure 3. RNF185/MBRL complex is not required for MC80-induced ERAD of TPSN.

(a) MC80-mediated TPSN degradation is inhibited upon p97 and proteasome inhibition. Flp-In T-Rex HEK293 cells expressing TPSN-sfGFP-3xHA were lentivirally transduced to express MC80-3xFLAG and were subsequently treated with the p97 inhibitor CB-5083 (2.5 μM) or the proteasome inhibitor Bortezomib (Btz; 500 nM) for 4 hours. TPSN-sfGFP-3xHA levels were assessed by flow cytometry (based on GFP fluorescence). Parental cells were included as control.

(b, c) RNF185/MBRL complex is dispensable for MC80-induced degradation of TPSN. RNF185 and MBRL were knocked out in cells expressing both TPSN-sfGFP-3xHA and MC80. Cells were then treated with the p97 inhibitor CB-5083 (2.5uM for 4 hours) and TPSN-sfGFP-3xHA levels were assessed by (b) flow cytometry (based on GFP fluorescence) or (c) immunoblotting using anti-HA antibody. MC80 was probed using FLAG-reactive antibody, and GAPDH was included as a loading control.

(d) MC80-induced degradation of TPSN depends on the ubiquitin conjugating enzyme UBE2G2 but not UBE2K, which assists RNF185/MBRL dependent ubiquitination. Ubiquitin conjugating enzymes UBE2G2 and UBE2K were depleted in MBRL knockout cells expressing both TPSN-sfGFP-3xHA and MC80. TPSN-sfGFP-3xHA levels were assessed by flow cytometry (based on GFP fluorescence).

(e) The ERAD ubiquitin ligases HRD1 and GP78 play redundant roles in MC80-induced degradation of TPSN. Ubiquitin ligases HRD1 and GP78 were depleted individually or simultaneously in cells expressing both TPSN-sfGFP-3xHA and MC80. TPSN-sfGFP-3xHA levels were assessed by flow cytometry (based on GFP fluorescence).

Supplementary Figure 4. Evolutionary conserved charge in transmembrane domain is a determinant for TPSN assembly and degradation.

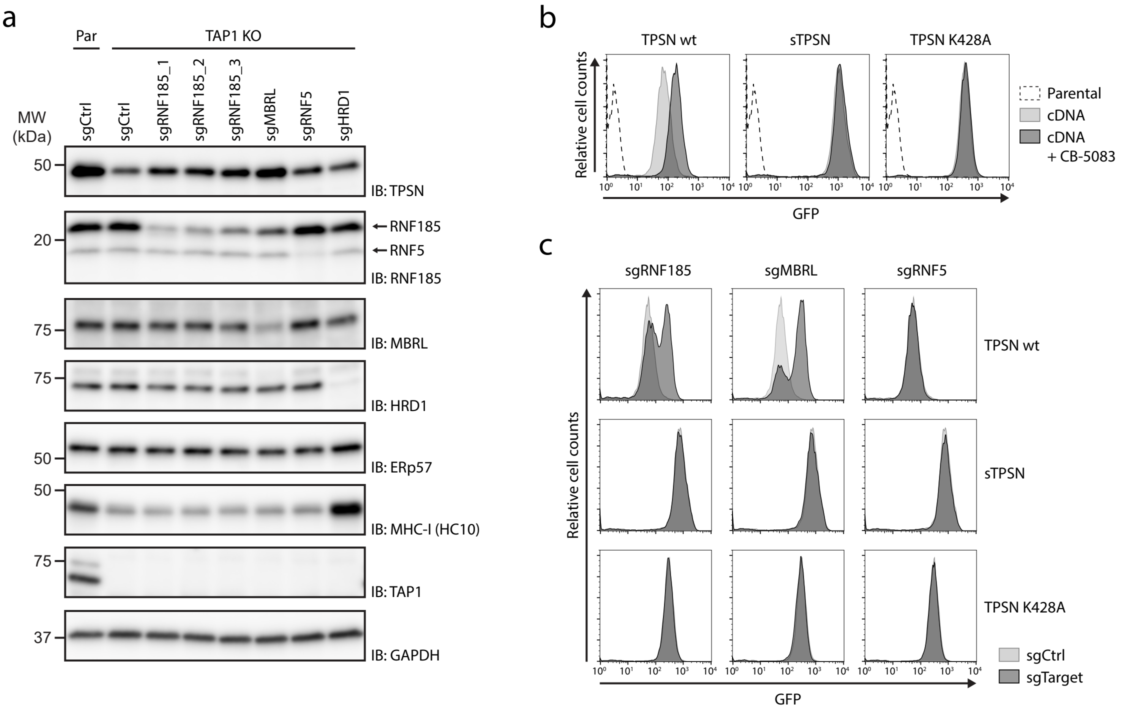

d

TMD sequence

TPSN	VGLFLSAFLLLGLF**K**ALGWAAVY
TCRαTM	NLSVIGF**R**ILLL**K**VAGFNLLMTL
CYP26A1TM	LPALLASALCTFVLPLLLFLAAI

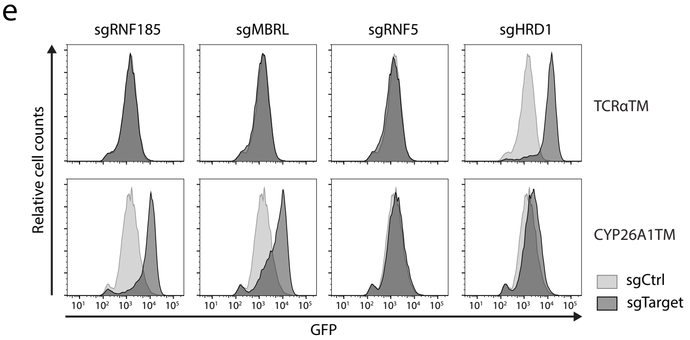

Supplementary Figure 4. TPSN assembly and degradation depends on evolutionary conserved charge in its transmembrane domain

(a) The RNF185/MBRL complex mediates TPSN degradation upon TAP1 knockout. TAP1 knockout cells were transfected with plasmids containing a non-targeting sgRNA or sgRNAs targeting RNF5, RNF185, or MBRL. Lysates from these cells were subjected to SDS-PAGE followed by immunoblotting with the indicated antibodies.

(b) TPSN TMD, in particular Lysine 428, is essential for degradation by the RNF185/MBRL complex. Cells expressing the indicated TPSN variants were depleted of RNF185, MBRL, and RNF5 using sgRNAs. TPSN-sfGFP-3xHA levels were assessed by flow cytometry (based on GFP fluorescence).

(c) Soluble TPSN (sTPSN) and TPSN K428A mutants are stable and no more degraded by ERAD. Flp-In T-Rex HEK293 cells expressing the indicated TPSN variants fused to sfGFP-3xHA were either left untreated or treated for 4 hours with the p97 inhibitor CB-5083 (2.5 µM) and analysed by flow cytometry (based on GFP fluorescence).

(d) Amino acid sequences of TPSN, TCRαTM, and CYP26A1TM transmembrane segments. Positive-charged residues are highlighted in bold red.

(e) Cells expressing TCRαTM- and CYP26A1TM-sfGFP-3xHA were depleted for RNF185, MBRL, RNF5 and HRD1 and subjected to flow cytometry for assessing GFP intensity.

Supplementary Table 1 - List of reagents used in this study

REAGENT or RESOURCE	SOURCE	IDENTIFIER
**Antibodies**		
Rabbit Monoclonal anti-Calnexin N-term (EPR3632)	Abcam	ab92573, RRID:AB_10563673
Rabbit Polyclonal anti-Calreticulin	Abcam	ab2907, RRID:AB_303402
Rabbit Monoclonal anti-RNF185 (EPR14070-94)	Abcam	ab181999, RRID:AB_2922962
Rabbit Monoclonal anti-TMUB1 (EPR14066)	Abcam	ab180586, RRID:AB_2922961
Rabbit Monoclonal anti-BiP clone (EPR4040(2))	Abcam	ab108613, RRID:AB_10859806
Mouse Monoclonal anti-TAPBP-R antibody (OTI1C9)	Abcam	ab236419
Rabbit Polyclonal anti-Membralin/TMEM259	Atlas Antibodies	HPA042669, RRID:AB_10794916
Rabbit Monoclonal anti-Tapasin (E6P2Z)	Cell Signaling Technology	66382
Rabbit Monoclonal anti-TAP1 (E4T4F)	Cell Signaling Technology	49671
Rabbit Monoclonal anti-TAP2 (E8G5I)	Cell Signaling Technology	25657
Rabbit Monoclonal anti-HRD1 (D3O2A)	Cell Signaling Technology	14773, RRID:AB_2798607
Rabbit Monoclonal anti-PDI (C81H6)	Cell Signaling Technology	3501, RRID:AB_2156433
Rabbit Monoclonal anti-Ubiquitin (E4I2J)	Cell Signaling Technology	43124, RRID:AB_2799235
Rabbit Monoclonal anti-Phospho-Stat1 (Tyr701) (D4A7)	Cell Signaling Technology	7649, RRID:AB_10950970
Rabbit Polyclonal anti-ERp57	Genetex	GTX113719, RRID:AB_10720538
Rabbit Polyclonal anti-TMUB2	ProteinTech	28044-1-AP, RRID:AB_2881045
Rabbit Polyclonal anti-CYP51A1	ProteinTech	13431-1-AP, RRID:AB_2088571
Mouse Monoclonal anti-GAPDH (1E6D9)	ProteinTech	60004-1-Ig, RRID:AB_2107436
Rat Monoclonal anti-HA (3F10)	Roche	11867423001, RRID:AB_390918

Rat Monoclonal anti-Tubulin (YOL1/34)	Santa Cruz Biotechnology	sc-53030, RRID:AB_2272440
Mouse Monoclonal anti-FLAG-HRP (M2)	Merck Life Science UK Limited	A8592, RRID:AB_439702
Mouse Monoclonal anti-MHC-I HC10	[1]	
Mouse Monoclonal anti-MHC-I HCA2	[2]	
Mouse Monoclonal anti-Tapasin PaStaI	[3]	
Mouse Monoclonal anti-Tapasin PaStaII	[4]	
Peroxidase AffiniPure Donkey Anti-Mouse IgG (H+L)	Jackson ImmunoResearch	715-035-150, RRID:AB_2340770
Peroxidase IgG Fraction Monoclonal Mouse Anti-Rabbit IgG, light chain specific	Jackson ImmunoResearch	211-032-171, RRID:AB_2339149
Peroxidase AffiniPure Goat Anti-Rat IgG, light chain specific	Jackson ImmunoResearch	112-035-175, RRID:AB_2338140
Peroxidase AffiniPure Goat Anti-Mouse IgG, light chain specific	Jackson ImmunoResearch	115-035-174, RRID:AB_2338512
Mouse Monoclonal anti-MHC-I W6/32 APC-conjugated	BioLegend	311410, RRID:AB_314879
APC Mouse IgG2a, κ Isotype Ctrl Antibody	BioLegend	400220
Anti-human CD14 FITC-conjugated	Immuno Tools GmbH	21270143
Anti-human CD45 FITC-conjugated	Immuno Tools GmbH	21270453
Mouse IgG1 control FITC-conjugated	Immuno Tools GmbH	21335013
**Chemicals, Peptides, and Recombinant Proteins**		
DMEM medium	Merck Life Science UK Limited	D6429
Fetal Calf Serum	Merck Life Science UK Limited	F9665
L-Glutamine (200 mM)	Gibco (Thermo Fisher Scientific)	25030024
Penicillin-Streptomycin (10,000 U/mL)	Gibco (Thermo Fisher Scientific)	15140122
Zeocin	Invitrogen (Thermo Fisher Scientific)	R25001

Puromycin	Gibco (Thermo Fisher Scientific)	A1113803
Doxycycline	Merck Life Science UK Limited	D9891
Cycloheximide	Merck Life Science UK Limited	C7698
TransIT LT1	Mirus Bio LLC	MIR 2305
OptiMEM	Gibco (Thermo Fisher Scientific)	31985062
Polybrene	Santa Cruz Biotechnology	28728-55-4
Papain	Worthington	LS003126
Fetal Bovine Serum	Nacalai USA	174012-500ML-BULK
Penicillin/streptomycin	Thermo Fisher Scientific	15140122
Matrigel	Corning	356237
Trypsin	Thermo Fisher Scientific	15090046
Poly-L-lysine	Merck Life Science UK Limited	P8920
cOmplete EDTA-free protease inhibitor cocktail	Roche	5056489001
Benzonase	Merck Life Science UK Limited	E1014
N-Ethylmaleimide (NEM)	Merck Life Science UK Limited	E3876
1,4-Dithiothreitol (DTT)	Merck Life Science UK Limited	D9779
DMNG	Anatrace	NG322
GDN	Anatrace	GDN101
CB-5083	Selleckchem	S8101
Bortezomib	Cell Signaling	2204
OXE8 medium	[5]	
FGF-2 basic 145aa	Bio-Techne (R&D Systems)	4114-TC-01M
TGF-beta	Peprotech	AF-100-21C
Accutase	Gibco	A11105-01

Human IgG	Merck Life Science UK Limited	I8640-100MG
Geltrex	Gibco (Thermo Fisher Scientific)	A1413302
Rho kinase inhibitor (ROCKi) Y-27632	Abcam	ab120129
Alt-R™ AsCas12a (Cpf1) Ultra	IDT	10001272
Advanced DMEM	Gibco (Thermo Fisher Scientific)	12491
GlutaMAX	Gibco (Thermo Fisher Scientific)	35050
2-Mercaptoethanol (50mM)	Gibco (Thermo Fisher Scientific)	31350
Gelatin 2%	Merck Life Science UK Limited	G1393-100ML
KnockOut DMEM	Gibco (Thermo Fisher Scientific)	10829
KnockOut Serum replacement	Gibco (Thermo Fisher Scientific)	10828
Non-Essential Amino Acids	Gibco (Thermo Fisher Scientific)	11140
bFGF	Bio-Techne (R&D Systems)	234-FSE/CF
**Critical Commercial Assays**		
S-Trap micro spin columns	Protify	CO2-micro-80
TMT10plex™ Isobaric Labeling kit	Thermo Fisher Scientific	90406/A34807, lot UG287488
Pierce™ Peptide Desalting Spin Columns	Thermo Fisher Scientific	89851
Pierce™ Anti-HA Magnetic Beads	Thermo Fisher Scientific	88837
Pierce™ Protein G Magnetic Beads	Thermo Fisher Scientific	88848
Western Lightning ECL Pro	Perkin Elmer	NEL121001EA
**Deposited Data**		
Proteomics	This study; PRIDE Data Set	PXD048728; https://www.ebi.ac.uk/pride/archive/projects/PXD048728
Original western blot images	This study; Mendeley Data Set	https://doi.org/10.17632/vfd47jgr8t

**Experimental Models: Cell Lines & Mouse strains**		
Flp-In™ T-REx™ 293 Cell Line	Invitrogen (Thermo Fisher Scientific)	R78007
Lenti-X™ 293T Cell Line	Clontech (Takara Bio)	632180
U2OS	ECACC	92022711
THP-1	ECACC	88081201
hIPSC line SFC840-03-03	[6]	
B6;129-Tmem259$^{tm1.1Itl}$/J mouse	The Jackson Laboratory	016574
FVB/N-Tmem163$^{Tg(ACTB\text{-}cre)2Mrt}$/J mouse	The Jackson Laboratory	003376
**sgRNA/crRNA sequences**		
RNF185 sgRNA: gAATGGCGCTGGCGAGAGCGG	[7]	
MBRL sgRNA: GAAGAACTCGAAGAGACGG	[7]	
RNF5 sgRNA: GCCAAATCGCGAGCGGGGCG	[7]	
HRD1 sgRNA: gACACCAGTTCTACCCCACTG	[7]	
TPSN sgRNA: GGTGCACTGCTGTTGCGCCA	This study	
TMUB1 sgRNA: GTTGCTGTGAACCCCGTGCT	[7]	
TMUB2 sgRNA: GCTACCGCTGTCTGCTACGT	[7]	
UBE2K sgRNA: GCTGCAATGACTCTCCGCA	[7]	
UBE2G2 sgRNA: GGAGAAGATCCTGCTGTCGG	[7]	
UBE2J1 sgRNA: gCCATCAAAATCGGAGTCTGG	[7]	
UBE2J2 sgRNA: GTTGCACTTAAACCTCCCGT	[7]	
TAP1 sgRNA: GGGGTCCTCAGGGCAACGGT	[8]	
GP78 sgRNA: GTCTCTCACTCACTCGAAGA	[7]	
AsCas12a RNF185 crRNA:<br>#1: GAGTGCAACATCTGCTTGGAC<br>#2: CAAACTTGGCAATGTGCTCTC	This study	
AsCas12a MBRL crRNA:<br>#1: TGCTCTTCGTCCTGGCCTACA<br>#2: AGGGACCTGGCATGCAGACAA	This study	
**Recombinant DNA**		

pcDNA5-FRT-TO plasmid	Thermo Fisher Scientific	V652020
pOG44 Flp-Recombinase Expression Vector	Thermo Fisher Scientific	V600520
Lentiviral cDNA expression vectors	[9]	
Lentiviral CRISPR/Cas9 vector	[9]	
pMD2.G	Addgene	12259
psPAX2	Addgene	12260
**Software**		
MaxQuant, version 1.6.3.4; 1.6.10.43	MaxQuant	https://www.maxquant.org/
Perseus software, version 1.5.5.3; 1.6.14.0	MaxQuant	https://maxquant.net/perseus/
Image studio software Li-Cor	Li-Cor	https://www.licor.com/bio/image-studio-lite/
FlowJo 10.8	FlowJo, LLC	https://www.flowjo.com/
GraphPad Prism	GraphPad	https://www.graphpad.com/scientific-software/prism/
R	3.6.2	https://www.r-project.org/

References

1. Stam, N. J., Spits, H. & Ploegh, H. L. Monoclonal antibodies raised against denatured HLA-B locus heavy chains permit biochemical characterization of certain HLA-C locus products. J Immunol 137, 2299–306 (1986).

2. Stam, N. J., Vroom, T. M., Peters, P. J., Pastoors, E. B. & Ploegh, H. L. HLA-A- and HLA-B-specific monoclonal antibodies reactive with free heavy chains in western blots, in formalin-fixed, paraffin-embedded tissue sections and in cryo-immuno-electron microscopy. Int Immunol 2, 113–125 (1990).

3. Dick, T. P., Bangia, N., Peaper, D. R. & Cresswell, P. Disulfide bond isomerization and the assembly of MHC class I-peptide complexes. Immunity 16, 87–98 (2002).

4. Dong, G., Wearsch, P. A., Peaper, D. R., Cresswell, P. & Reinisch, K. M. Insights into MHC class I peptide loading from the structure of the tapasin-ERp57 thiol oxidoreductase heterodimer. Immunity 30, 21–32 (2009).

5. Vaughan-Jackson, A. et al. Differentiation of human induced pluripotent stem cells to authentic macrophages using a defined, serum-free, open-source medium. Stem Cell Reports 16, 1735–1748 (2021).

6. Fernandes, H. J. R. et al. ER Stress and Autophagic Perturbations Lead to Elevated Extracellular α-Synuclein in GBA-N370S Parkinson’s iPSC-Derived Dopamine Neurons. Stem Cell Reports 6, 342–356 (2016).

7. van de Weijer, M. L. et al. Quality Control of ER Membrane Proteins by the RNF185/Membralin Ubiquitin Ligase Complex. Mol Cell 79, 768-781.e7 (2020).

8. Praest, P. et al. The influence of TAP1 and TAP2 gene polymorphisms on TAP function and its inhibition by viral immune evasion proteins. Mol Immunol 101, 55–64 (2018).

9. van de Weijer, M. L. M. L. et al. A high-coverage shRNA screen identifies TMEM129 as an E3 ligase involved in ER-associated protein degradation. Nat Commun 5, 3832 (2014).
