# Supplementary material for: Tapasin assembly surveillance by the RNF185/Membralin ubiquitin ligase complex regulates MHC-I surface expression
Source: Nat Commun. 2024 Oct 1;15:8508. doi: 10.1038/s41467-024-52772-x (PMC11445256; doi:10.1038/s41467-024-52772-x)
Supplement: Supplementary file 3 — Reporting Summary [file 41467_2024_52772_MOESM3_ESM.pdf]

nature portfolio

Corresponding author(s): Pedro Carvalho

Last updated by author(s): 2024/03/08

Reporting Summary

Nature Portfolio wishes to improve the reproducibility of the work that we publish. This form provides structure for consistency and transparency in reporting. For further information on Nature Portfolio policies, see our Editorial Policies and the Editorial Policy Checklist.

Statistics

For all statistical analyses, confirm that the following items are present in the figure legend, table legend, main text, or Methods section.

n/a	Confirmed	
☐	☒	The exact sample size (*n*) for each experimental group/condition, given as a discrete number and unit of measurement
☐	☒	A statement on whether measurements were taken from distinct samples or whether the same sample was measured repeatedly
☐	☒	The statistical test(s) used AND whether they are one- or two-sided<br>*Only common tests should be described solely by name; describe more complex techniques in the Methods section.*
☒	☐	A description of all covariates tested
☒	☐	A description of any assumptions or corrections, such as tests of normality and adjustment for multiple comparisons
☐	☒	A full description of the statistical parameters including central tendency (e.g. means) or other basic estimates (e.g. regression coefficient) AND variation (e.g. standard deviation) or associated estimates of uncertainty (e.g. confidence intervals)
☐	☒	For null hypothesis testing, the test statistic (e.g. *F*, *t*, *r*) with confidence intervals, effect sizes, degrees of freedom and *P* value noted<br>*Give P values as exact values whenever suitable.*
☒	☐	For Bayesian analysis, information on the choice of priors and Markov chain Monte Carlo settings
☒	☐	For hierarchical and complex designs, identification of the appropriate level for tests and full reporting of outcomes
☒	☐	Estimates of effect sizes (e.g. Cohen's *d*, Pearson's *r*), indicating how they were calculated

Our web collection on statistics for biologists contains articles on many of the points above.

Software and code

Policy information about availability of computer code

Data collection	BD FACSDiva
Data analysis	MaxQuant v1.6.3.4, v1.6.10.43; Perseus v1.5.5.3, v1.6.14.0; R v3.6.2; FlowJo 10.8; Li-Cor Image Studio Lite v5.2, GraphPad Prism v10

For manuscripts utilizing custom algorithms or software that are central to the research but not yet described in published literature, software must be made available to editors and reviewers. We strongly encourage code deposition in a community repository (e.g. GitHub). See the Nature Portfolio guidelines for submitting code & software for further information.

Data

Policy information about availability of data

All manuscripts must include a data availability statement. This statement should provide the following information, where applicable:

- Accession codes, unique identifiers, or web links for publicly available datasets
- A description of any restrictions on data availability
- For clinical datasets or third party data, please ensure that the statement adheres to our policy

The Proteomics data have been deposited to the PRIDE proteomics repository with the dataset identifier PXD048728.
The western blotting data generated during this study are available at Mendeley Data (https://data.mendeley.com/preview/vfd47jgr8t?a=20a1c83c-4c9f-42e7-bf1e-fa7b640cdfad)

Research involving human participants, their data, or biological material

Policy information about studies with human participants or human data. See also policy information about sex, gender (identity/presentation), and sexual orientation and race, ethnicity and racism.

Reporting on sex and gender	N/A
Reporting on race, ethnicity, or other socially relevant groupings	N/A
Population characteristics	N/A
Recruitment	N/A
Ethics oversight	N/A

Note that full information on the approval of the study protocol must also be provided in the manuscript.

Field-specific reporting

Please select the one below that is the best fit for your research. If you are not sure, read the appropriate sections before making your selection.

☒ Life sciences ☐ Behavioural & social sciences ☐ Ecological, evolutionary & environmental sciences

For a reference copy of the document with all sections, see nature.com/documents/nr-reporting-summary-flat.pdf

Life sciences study design

All studies must disclose on these points even when the disclosure is negative.

Sample size	Mouse sample size was determined by the amount of protein material (from astrocytes ex vivo) required for TMT MassSpectrometry analysis.
Data exclusions	No data was excluded from the analyses.
Replication	All experiments, except mass spectrometry, were independently repeated at least 3 times. For the mouse experiment, 5 parental and 5 MBRL KO pups were used.
Randomization	Randomization was not applicable and not applied in this study.
Blinding	Blinding was not applicable and not applied in this study.

Reporting for specific materials, systems and methods

We require information from authors about some types of materials, experimental systems and methods used in many studies. Here, indicate whether each material, system or method listed is relevant to your study. If you are not sure if a list item applies to your research, read the appropriate section before selecting a response.

Materials & experimental systems

n/a	Involved in the study	
☐	☒	Antibodies
☐	☒	Eukaryotic cell lines
☒	☐	Palaeontology and archaeology
☐	☒	Animals and other organisms
☒	☐	Clinical data
☒	☐	Dual use research of concern
☒	☐	Plants

Methods

n/a	Involved in the study	
☒	☐	ChIP-seq
☐	☒	Flow cytometry
☒	☐	MRI-based neuroimaging

Antibodies

Antibodies used	Rabbit Monoclonal anti-Calnexin N-term (EPR3632) Abcam ab92573, RRID:AB_10563673<br>Rabbit Polyclonal anti-Calreticulin Abcam ab2907, RRID:AB_303402<br>Rabbit Monoclonal anti-RNF185 (EPR14070-94) Abcam ab181999, RRID:AB_2922962

Rabbit Monoclonal anti-TMUB1 (EPR14066) Abcam ab180586, RRID:AB_2922961
Rabbit Monoclonal anti-BiP clone (EPR4040(2)) Abcam ab108613, RRID:AB_10859806
Mouse Monoclonal anti-TAPBP-R antibody (OTI1C9) Abcam ab236419
Rabbit Polyclonal anti-Membralin/TMEM259 Atlas Antibodies HPA042669, RRID:AB_10794916
Rabbit Monoclonal anti-Tapasin (E6P2Z) Cell Signaling Technology 66382
Rabbit Monoclonal anti-TAP1 (E4T4F) Cell Signaling Technology 49671
Rabbit Monoclonal anti-TAP2 (E8G5I) Cell Signaling Technology 25657
Rabbit Monoclonal anti-HRD1 (D3O2A) Cell Signaling Technology 14773, RRID:AB_2798607
Rabbit Monoclonal anti-PDI (C81H6) Cell Signaling Technology 3501, RRID:AB_2156433
Rabbit Monoclonal anti-Ubiquitin (E4I2J) Cell Signaling Technology 43124, RRID:AB_2799235
Rabbit Monoclonal anti-Phospho-Stat1 (Tyr701) (D4A7) Cell Signaling Technology 7649, RRID:AB_10950970
Rabbit Polyclonal anti-ERp57 Genetex GTX113719, RRID:AB_10720538
Rabbit Polyclonal anti-TMUB2 ProteinTech 28044-1-AP, RRID:AB_2881045
Rabbit Polyclonal anti-CYP51A1 ProteinTech 13431-1-AP, RRID:AB_2088571
Mouse Monoclonal anti-GAPDH (1E6D9) ProteinTech 60004-1-Ig, RRID:AB_2107436
Rat Monoclonal anti-HA (3F10) Roche 11867423001, RRID:AB_390918
Rat Monoclonal anti-Tubulin (YOL1/34) Santa Cruz Biotechnology sc-53030, RRID:AB_2272440
Mouse Monoclonal anti-FLAG-HRP (M2) Merck Life Science UK Limited A8592, RRID:AB_439702
Mouse Monoclonal anti-MHC-I HC10 Stam et al 1986 J Immunology
Mouse Monoclonal anti-MHC-I HCA2 Stam et al 1990 Int Immunology
Mouse Monoclonal anti-Tapasin PaStaI Dick et al 2002 Immunity
Mouse Monoclonal anti-Tapasin PaStaII Dong et al 2009 Immunity
Peroxidase AffiniPure Donkey Anti-Mouse IgG (H+L) Jackson ImmunoResearch 715-035-150, RRID:AB_2340770
Peroxidase IgG Fraction Monoclonal Mouse Anti-Rabbit IgG, light chain specific Jackson ImmunoResearch 211-032-171, RRID:AB_2339149
Peroxidase AffiniPure Goat Anti-Rat IgG, light chain specific Jackson ImmunoResearch 112-035-175, RRID:AB_2338140
Peroxidase AffiniPure Goat Anti-Mouse IgG, light chain specific Jackson ImmunoResearch 115-035-174, RRID:AB_2338512
Mouse Monoclonal anti-MHC-I W6/32 APC-conjugated BioLegend 311410, RRID:AB_314879
APC Mouse IgG2a, κ Isotype Ctrl Antibody BioLegend 400220
Anti-human CD14 FITC-conjugated Immuno Tools GmbH 21270143
Anti-human CD45 FITC-conjugated Immuno Tools GmbH 21270453
Mouse IgG1 control FITC-conjugated Immuno Tools GmbH 21335013

Validation

Primary antibodies were validated by including negative controls, such as isotype or KO samples, and/or by including positive controls, such as overexpression samples. The correct size of the bands on western blots were checked using markers. Antibody validation information from the manufacturer's websites was also consulted.

Eukaryotic cell lines

Policy information about cell lines and Sex and Gender in Research

Cell line source(s)

U2OS and THP-1 cells were obtained from the ECACC. The Lenti-X 293T cell line for production of lentivirus was obtained from TakaraBio. Flp-In T-REx HEK293 cells were obtained from Invitrogen (Thermo Fischer Scientific). Human iPSC line SFC840-03-03 49 was obtained from EBiSC (https://ebisc.org/STBCi026-A).

Authentication

Authenticity was guaranteed by the supplier.

Mycoplasma contamination

Cells were routinely checked for mycoplasma in-house using Lonza MycoAlert mycoplasma detection kit

Commonly misidentified lines
(See ICLAC register)

N/A

Animals and other research organisms

Policy information about studies involving animals; ARRIVE guidelines recommended for reporting animal research, and Sex and Gender in Research

Laboratory animals

B6;129-Tmem259tm1.1Itl/J mouse, The Jackson Laboratory, 016574.
FVB/N-Tmem163Tg(ACTB-cre)2Mrt/J mouse, The Jackson Laboratory, 003376.

Wild animals

N/A

Reporting on sex

The sex of the animals was not selected for.

Field-collected samples

N/A

Ethics oversight

Ethical approval for the mouse experiment was provided by the ethical committee at the Sanford Burnham Prebys Medical Discovery Institute

Note that full information on the approval of the study protocol must also be provided in the manuscript.

Plants

Seed stocks	N/A
Novel plant genotypes	N/A
Authentication	N/A

Flow Cytometry

Plots

Confirm that:

☒ The axis labels state the marker and fluorochrome used (e.g. CD4-FITC).

☒ The axis scales are clearly visible. Include numbers along axes only for bottom left plot of group (a 'group' is an analysis of identical markers).

☒ All plots are contour plots with outliers or pseudocolor plots.

☒ A numerical value for number of cells or percentage (with statistics) is provided.

Methodology

Sample preparation	For surface staining of MHC-I, cells were detached using EDTA, resuspended in FACS buffer (2% FBS, 2 mM EDTA in PBS) and washed once. Cells were incubated with W6/32-APC (1:50; Biolegend #311410) for 1 hour at 4°C. Next, cells were washed twice in FACS buffer and directly analysed using a BD LSRFortessa X-20 flow cytometer. For fluorescence measurement of GFP, cells were trypsinized, resuspended in FACS buffer, and directly analysed on the BD X-20. FACS data was analysed using FlowJo v10.
Instrument	BD LSRFortessa™ X-20
Software	BD FACSDiva for collection. FlowJo v10 for analysis
Cell population abundance	N/A
Gating strategy	At least 10000 cells per sample were measured gated on the main population in the FSC/SSC plot.

☐ Tick this box to confirm that a figure exemplifying the gating strategy is provided in the Supplementary Information.
